# Effect of Water Regimen on Fruit Growth, Metabolomic Profile, and Postharvest Quality of ‘Hass’ Avocados

**DOI:** 10.3390/plants15121807

**Published:** 2026-06-11

**Authors:** Daniela Olivares, María E. Ulloa, José I. Covarrubias, Edgard Álvarez, Miguel Á. García-Rojas, Carolina Salazar, Rodrigo Candia, Reinaldo Campos-Vargas, Romina Pedreschi, Bruno G. Defilippi

**Affiliations:** 1 Instituto de Investigaciones Agropecuarias, INIA-La Platina, Santiago 8831314, Chile; olivaresdaniela@gmail.com (D.O.); edgar.alvarez@inia.cl (E.Á.); carolina.salazar@inia.cl (C.S.); rodrigo.candia@inia.cl (R.C.); 2 Facultad de Ciencias Agronómicas, Universidad de Chile, Santiago 8820808, Chile; jcovarru@uchile.cl (J.I.C.); reinaldocampos@uchile.cl (R.C.-V.); 3 Facultad de Ciencias Agronómicas y de los Alimentos, Pontificia Universidad Católica de Valparaíso, Quillota 2260000, Chile; romina.pedreschi@pucv.cl; 4 Millennium Institute Center for Genome Regulation, Santiago 8331150, Chile

**Keywords:** water regimen, mineral content, global quality, avocado, postharvest

## Abstract

Preharvest climatic conditions and irrigation management are decisive determinants of avocado postharvest performance. Avocado trees are highly susceptible to the water regimen, conditions that disrupt carbon assimilation, mineral nutrient uptake, and biomass partitioning. This study evaluated the effects of deficit irrigation imposed during early stages of fruit growth, coinciding with active cell division, on fruit development and postharvest quality of ‘Hass’ avocado. Deficit and excess irrigation induced physiological stress, reducing stem water potential (≈−1 MPa) and altering photochemical efficiency, while F_V_/F_M_ remained unaffected. Fruit growth was strongly affected, with weight reductions of up to 26% during development and 22% at harvest under severe deficit, resulting in fruits becoming more yellowish-green. In contrast, excessive irrigation promoted larger fruit with darker green skin, with delayed maturation. Metabolomic revealed that the fruit developmental stage was the main driver of metabolic variation, while irrigation effects were minor and stage-dependent, limited to osmotic-related metabolites such as GABA. These findings indicate that early-season water imbalances primarily affect fruit growth through changes in water relations rather than metabolic reprogramming, highlighting the importance of precise irrigation management during critical developmental stages. Fine-tuning water supply during early developmental stages is a strategic tool for optimizing fruit size and postharvest quality in avocado.

## 1. Introduction

The postharvest quality of avocado is strongly influenced by climatic conditions and orchard management practices, which determine the physiological status of the tree and subsequent fruit development [1,2]. Under current climate change scenarios, avocado production systems in Chile are highly vulnerable to prolonged droughts, rising temperatures, and decreasing water availability [3,4]. This environmental variability alters cellular functions, affecting metabolic processes, hormonal regulation, and crop physiology. Consequently, these alterations compromise fruit quality attributes, including appearance, flavor, and nutritional content [5,6].

In Chile, avocado production is primarily concentrated in the central region, which features a temperate Mediterranean climate that minimizes frost risk but includes long, hot, and dry summers that necessitate irrigation. In this region, water availability is the primary limiting factor for agricultural production, and climate change is expected to exacerbate this constraint through increased temperatures, reduced rainfall, and more frequent drought events [7]. The flowering period lasts up to three months (September–November), and fruit set occurs immediately afterward, followed by a phase of intensive fruit growth. The first stage of fruit development, spanning up to 6 to 8 weeks post-fruit set, is critical regarding water availability and calcium uptake, both of which significantly influence fruit quality and postharvest performance [8]. Fruits can remain on the tree for 400 days or longer (e.g., in orchards located close to the coast), extending far beyond the time required to reach physiological maturity and the capacity to ripen [9]. Harvest can begin as early as September and extend for several months until February [6]. This extended on-tree period increases the exposure of developing fruit to environmental fluctuations, particularly variations in water availability, which may exert cumulative and long-lasting effects on fruit physiology and postharvest performance [9].

Recent studies have shown that deficit and excess irrigation alter plant growth and physiological performance, highlighting the high sensitivity of avocado to water stress [10]. Under deficit irrigation, reductions in stomatal conductance, leaf water potential, and photosynthetic activity are commonly observed, leading to decreased carbon assimilation and impaired growth [11]. Because calcium (Ca) is poorly mobile in the phloem, its accumulation in fruit is inherently limited and highly sensitive to changes in plant water status [12]. Low fruit Ca concentrations have been linked to mesocarp softening, exocarp browning, and the development of physiological disorders—such as lenticel damage and black spot—ultimately reducing postharvest quality and marketability [13,14]. Conversely, excess water reduces oxygen availability in the rhizosphere, inducing hypoxic or anoxic conditions that compromise root respiration, nutrient uptake, and hydraulic conductivity [15]. Importantly, these stress responses not only affect vegetative growth but also directly impact fruit development and composition, particularly when they occur during critical phenological stages. In this regard, irrigation strategies based on plant water status and soil moisture monitoring have been shown to improve water use efficiency without compromising yield. Thus, they represent a key tool for sustainable avocado production under water-limited conditions [16].

Despite its importance, it remains unclear how temporary water fluctuations during the early stages of fruit development—a period of intense cell division—affect the long-term physiological trajectory and final postharvest performance of ‘Hass’ avocados. Therefore, this study aimed to evaluate the effects of deficit and excess irrigation applied during early fruit development on plant physiological status, fruit growth dynamics, and postharvest quality. By examining these relationships, we aim to provide strategic insights to optimize irrigation management and ensure fruit marketability amid the increasing constraints on Mediterranean production systems.

## 2. Results

Different irrigation regimes were applied based on the reference evapotranspiration (ETo). The treatments were as follows: T1 (control) = irrigation with amounts of water equal to 100% ETo; T2 = irrigation with amounts of water equal to 40% ETo; T3 = irrigation with amounts of water equal to 60% ETo; T4 = irrigation with amounts of water equal to 120% ETo.

### 2.1. Environmental Conditions

**Figure 1:**
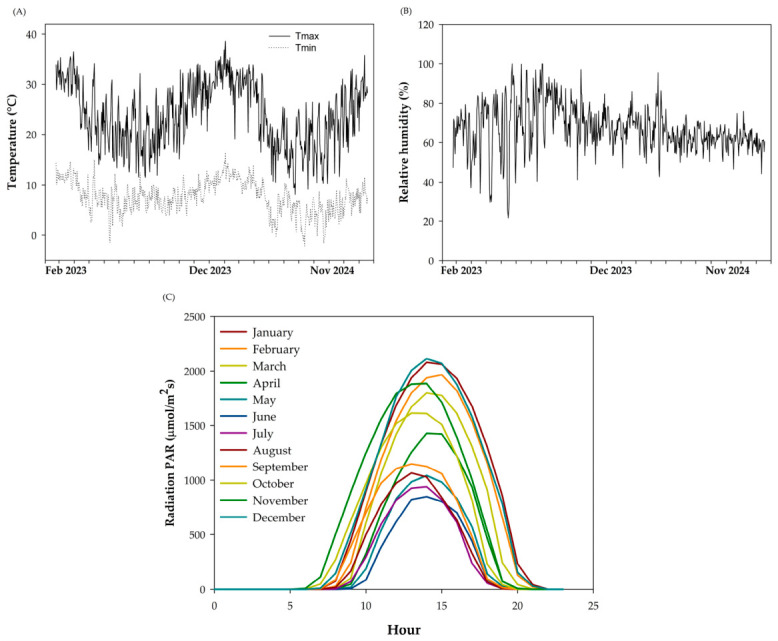
Environmental conditions for 2023–2024. (**A**) Mean air temperature, maximum and minimum; (**B**) relative humidity; and (**C**) radiation PAR profile per month (mean of 2 years).

The average monthly values of air temperature, relative humidity (RH), and photosynthetically active radiation (PAR) recorded during the experimental period (2023–2024) are shown in Figure 1. The average annual maximum air temperature (Tmax, °C) was 24.0 ± 0.3 °C. The highest Tmax values were recorded during December 2023 and March 2024, with a peak of 38.6 °C in February 2024. These months coincided with critical phenological stages of fruit development, including fruit set and the first phase of fruit growth. For the winter season (June to September), the average minimum air temperature (Tmin, °C) was 7.6 ± 0.1 °C, and nine below-zero temperature events were recorded, with a minimum temperature of −2.1 °C in July (2023). The mean RH was 67.2 ± 0.5%, and the highest PAR value (mean of two years) was observed between 14:00 and 15:00 h, with a maximum value of 1890.9 ± 63.1 µmol/m^2^s in summer and 1103.3 ± 73.2 µmol/m^2^s in winter.

### 2.2. Physiological Status

#### 2.2.1. Soil and Plant Water Status

**Figure 2:**
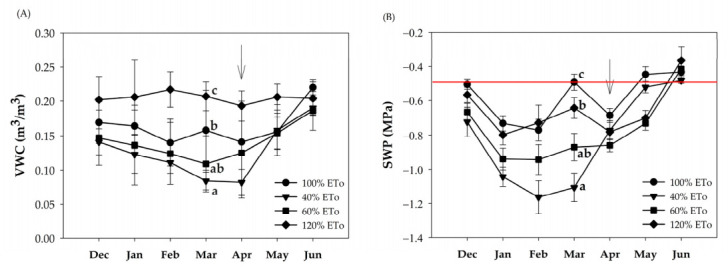
Soil and plant water status. (**A**) Volumetric water content (VWC; *n* = 24) in soil and (**B**) stem water potential (SWP; *n* = 18). The data represent the means ± standard deviations (SD). Different letters indicate significant differences among treatments at each evaluation time according to Tukey’s test (*p* < 0.05). The arrow indicates the moment when conventional orchard irrigation was reinstated. In (**B**), the red line corresponds to the optimal value (−0.5 MPa).

The irrigation regimes were applied from January to March, during early-stage development, a period corresponding to active fruit cell division and high calcium uptake and translocation. These processes are critical for subsequent postharvest performance. The temporal variation in volumetric water content (VWC) from the onset of water deficit until three months after the end of the irrigation treatments is shown in Figure 2A. As expected, the 120% ETo treatment resulted in the highest soil water content throughout the deficit period. In contrast, the severe deficit treatment resulted in markedly lower VWC values, reaching approximately half of those observed under overirrigation. Once the irrigation was restored to standard orchard conditions in April, the VWC values progressively converged, and no clear differences among the treatments were observed. Stem water potential (SWP) was measured at midday on several dates during the seasons for each irrigation treatment (Figure 2B). The values observed at the start of the experimental period were similar. At the beginning of the experimental period, potential values below the optimum (−0.5 MPa) reported for avocados were observed [17]. Notably, the orchard was located in a historically drought-affected area. During the irrigation treatments, SWP decreased significantly in the deficit treatments, reaching values close to −1.0 MPa under severe water restriction.

In contrast, the 100% ETo (control) and 120% ETo treatments maintained higher SWP values, generally above −0.8 MPa. By the end of the treatment period, significant differences among treatments were detected, with the deficit (40% and 60% ETo) and 120% ETo overirrigated treatments showing SWP values below the reported optimum. In contrast, only the control treatment remained close to −0.5 MPa. Following the restoration of irrigation, SWP values gradually increased, but differences among treatments diminished. Three months after the end of the irrigation treatments, SWP values converged across treatments and exceeded the −0.5 MPa threshold, indicating a recovery of plant water status.

#### 2.2.2. Photosynthetic Efficiency

**Figure 3:**
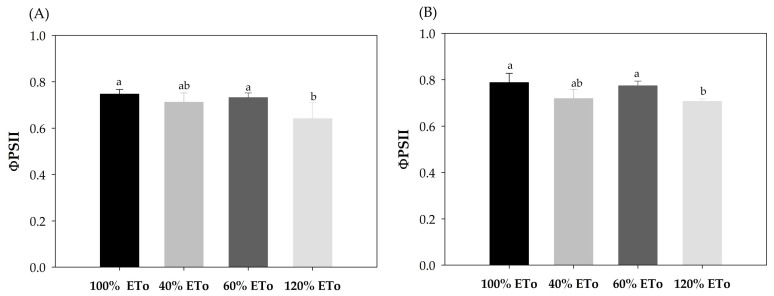
Maximum and effective quantum efficiency of photosystem II (ΦPSII) in avocado leaves (*Persea americana* cv. ‘Hass’) under different irrigation treatments: 100% ETo, 40% ETo, 60% ETo, and 120% ETo. (**A**) Effective quantum efficiency of ΦPSII during the irrigation regime and (**B**) effective quantum efficiency of ΦPSII after the end of the irrigation regime. The bars represent the means ± SD (*n* = 9). Different letters indicate significant differences among treatments at each evaluation time according to Tukey’s test (*p* < 0.05).

The effective quantum yield of the photosystem (ΦPSII) and the maximum quantum yield of photosystem II (F_V_/F_M_) were evaluated as indicators of plant physiological status. One month after the initiation of the irrigation regimes, there was a significant difference in the levels of ΦPSII (Figure 3A). The 100% and 60% ETo treatments had the highest ΦPSII values (0.732 and 0.729, respectively). In contrast, 120% ETo showed significantly lower ΦPSII (0.641), suggesting a reduction in photochemical performance under light conditions. The 40% ETo displayed intermediate values (0.712); however, according to post hoc analysis, 120% ETo differed significantly only from 100% and 60% ETo, while no significant differences were observed between 100%, 40%, and 60% ETo, or between 40% and 120% ETo. Despite these differences in ΦPSII, F_V_/F_M_ values did not differ significantly across irrigation regimes (ranging from 0.75 to 0.8 for all conditions) and remained within a relatively high range, indicating that no major damage to the photosynthetic apparatus occurred. Before harvest (1 month before), a similar pattern was observed for ΦPSII, but the values were higher in all treatments (Figure 3B). The F_V_/F_M_ did not differ significantly among treatments (mean value of 0.8), suggesting that the stress imposed did not cause structural damage to PSII.

### 2.3. Leaf Characteristics

As previously mentioned, the results obtained for VWC, SWP, and ΦPSII indicated that the different irrigation regimes affected the physiological status and photosynthetic efficiency of avocado plants. On this basis, the nutritional content (N, P, K, Ca, Mg, Cu, Fe, Mn, Zn, Na, and B), stomatal density, and leaf chlorophylls content were evaluated. The nutritional content of leaves is ideally determined prior to autumn sprouting. Therefore, after the treatments were applied, a nutritional analysis of the leaves was conducted. Slight differences were obtained among the mineral contents for the treatments; however, all values fell within the optimal range (Table 1), indicating that short-term water stress did not affect the overall nutritional balance of the leaves. In addition, no differences in stomatal number or chlorophyll content were observed among the treatments (Supplementary Materials).

### 2.4. Fruit Characteristics

#### 2.4.1. Quality Attributes During Fruit Development

**Figure 4:**
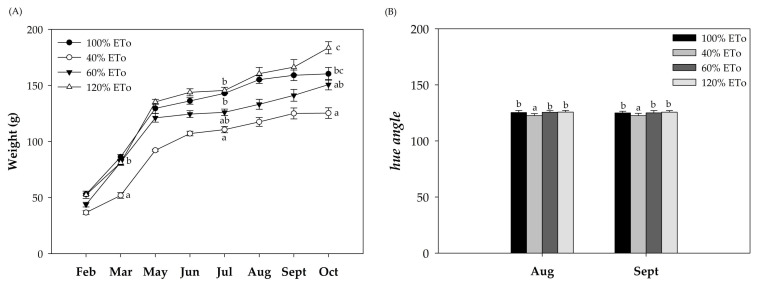
Fruit growth and color parameter during the development of avocado fruit subjected to different water stress treatments. (**A**) Weight, (**B**) *hue angle* by *CIELab* color space. The data represent the means ± SD (*n* = 10). Different letters indicate significant differences among treatments at each evaluation time according to Tukey’s test (*p* < 0.05).

During fruit development, quality attributes associated with fruit size (weight, equatorial and polar diameter), color, dry matter, and nutritional content were evaluated. The progression of fruit weight during the treatments and after the irrigation regime until harvest is shown in Figure 4A. Avocados collected from the 40% ETo treatment were lighter throughout fruit development; these fruits had a 26.3% lower weight, compared to the 100% ETo treatment. Fruit epidermis color was analyzed, revealing that the color of the fruit from the lowest irrigation treatment (40% ETo) differed from that of the fruit from other treatments from August 2024 onwards. Figure 4B shows that the fruits from the 40% ETo treatment had lower values for the *hue angle* parameter (122.5 ± 1.8) compared with those from other treatments (125.5 ± 1.5), despite achieving level 1 on the hedonic scale (where 100% of the skin surface is green), which coincides with a more yellowish-green color.

**Figure 5:**
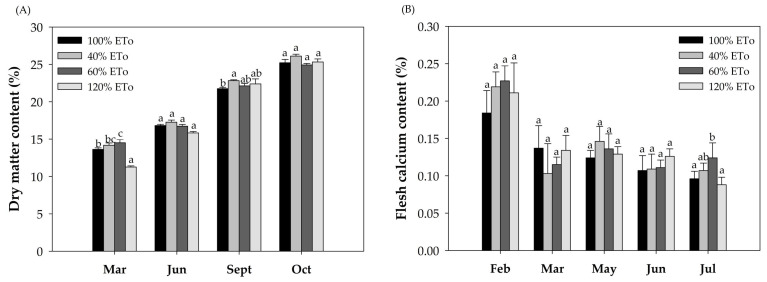
Dry matter content and calcium content during the development of avocado fruit subjected to different water stress treatments. (**A**) Dry matter content (%) and (**B**) flesh calcium content (%). The data represent the means ± SD (*n*= 10). Different letters indicate significant differences among treatments at each evaluation time according to Tukey’s test (*p* < 0.05).

Regarding the dry matter content, at the early stages of development, the fruits from the 120% ETo treatment showed lower dry matter (DM) values (Figure 5A).

Regarding fruit total calcium content, levels were determined in the mesocarp and epicarp, with higher values in the epicarp. As the effects of water deficit were similar for both tissues, only the levels in the flesh are presented (Figure 5B). At the time the irrigation regime was applied, similar calcium levels were observed across all treatments, with a maximum of 0.22%. By the end of the irrigation regime, calcium levels decreased, without showing differences among treatments.

#### 2.4.2. Physiological and Quality Parameters in Postharvest

At harvest, the weight of the fruit from the 40% ETo treatment was lower by 21.8%, compared with that of 100% ETo (Table 2). Regarding dry matter, the fruit was harvested with a DM content of 24–26%. Color development showed the same trend as that observed during fruit development. The fruit characteristics for each treatment at harvest are shown in Table 2.

**Figure 6:**
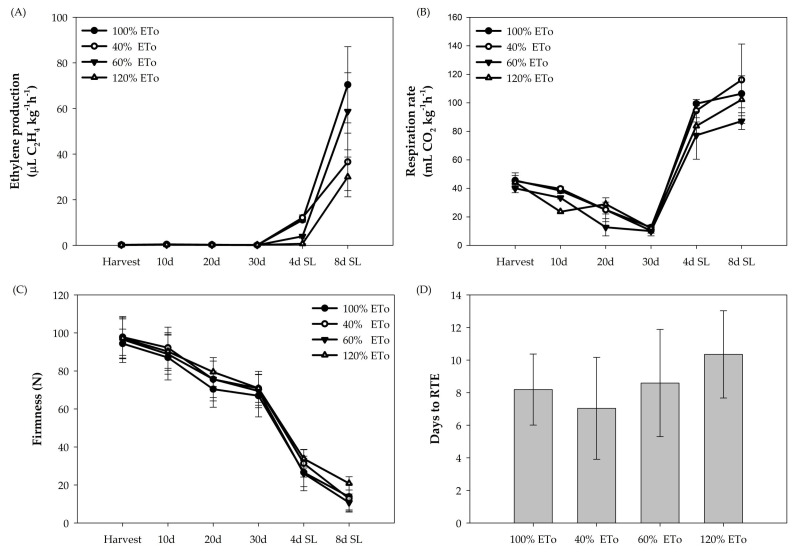
Physiological and quality parameters of avocado fruit subjected to different water-stress treatments during postharvest stage. (**A**) Ethylene production, (**B**) respiration rate, (**C**) firmness, and (**D**) days to ready-eat-time (RTE). The data represent the means ± SD (*n* = 15). For results obtained for all figures non-significant differences Tukey’s test (*p* < 0.05), among treatments.

The fruits were stored at 5 ± 0.5 °C under air atmosphere for 30 days. During this period, the physiological and maturity parameters were measured every 10 days (10 d, 20 d, and 30 d). At the end of cold storage (30 d), the fruits were placed under shelf-life (SL) conditions (20 ± 0.5 °C) until they reached the ready-to-eat (RTE) stage. The results are shown in Figure 6 and described below.

Ethylene production rates were very low in all fruits during cold storage (Figure 6A), ranging between 0.1 and 0.32 μL C_2_H_4_ kg^−1^h^−1^. During shelf life, the ethylene production rate in fruits from the 100% ETo treatment was observed to increase, reaching a maximum of 70.4 ± 16.7 μL C_2_H_4_ kg^−1^h^−1^. A similar pattern was observed for the other treatments, without showing statistical differences among treatments in each evaluation time. For the respiration rate, when fruits were exposed to 20 ± 0.5 °C, values tended to increase, as did ethylene production, reaching a maximum at consumption maturity (Figure 6B).

Firmness values during cold storage are presented in Figure 6C. After 20 days, the fruit from the 100% ETo treatment showed a firmness of 70.4 ± 9.5 N. The firmness of fruit from deficit irrigation (40% and 60% ETo) was nearly 76 N, whereas that from 120% ETo was 79.4 ± 7.6 N. By the end of cold storage, firmness values were around 70 N across all treatments; however, no fruits were at the firmness level preferred at consumption maturity (14 N). After 4 days at 20 ± 0.5 °C (SL), the fruit presented a firmness of 26–34 N. Under the 100%, 40%, and 60% ETo treatments, the fruit reached consumption maturity (below 14 N) after eight days at 20 ± 0.5 °C, whereas with the 120% ETo treatment, it took ten days (Figure 6D). Regarding physiological disorders, only vascular browning was observed during shelf-life storage. The 120% ETo treatment was the most affected, with a 4.2% incidence rate, but it showed signs of damage in the lowest severity range considered on the hedonic scale (level 2, 5 to 10% of the mesocarp is affected).

**Figure 7:**
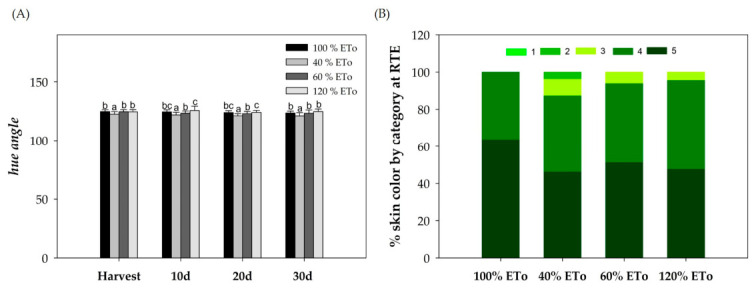
Skin color during cold storage for avocado harvested in 2024. (**A**) *hue angle* by *CIELab* color space, (**B**) skin color by category at ready-eat-time (RTE). For (**A**), data represent the means ± SD. Different letters indicate significant differences among treatments at each evaluation time according to Tukey’s test (*p* < 0.05).

The skin color of the fruits under all treatments did not change during cold storage. The fruit was green, corresponding to level 1 on the hedonic scale (where 100% of the skin surface is green). Regarding the color quality parameters, the color trend observed during the preharvest period was maintained during postharvest storage at 5 ± 0.5 °C. The *hue angle* values for fruit obtained from 40% ETo were lower (Figure 7A), indicating that the skin color was more yellowish-green than that in the other treatments.

At the RTE stage, all fruits under the 100% ETo treatment were at the highest commercial color level (4–5 on the scale). In contrast, under the 40% ETo treatment, only 87.5% of the fruits achieved this commercial purple/black coloration, with some fruits remaining at color level 2 (3.6%) (Figure 7B).

### 2.5. Metabolomics

Based on the results, metabolomic analyses were performed on fruit under 100% ETo and 40% ETo at the development and harvest stages. Metabolomic analysis was performed on mesocarp and epicarp samples (Supplementary Figures S1–S3). However, only the mesocarp results are shown.

**Figure 8:**
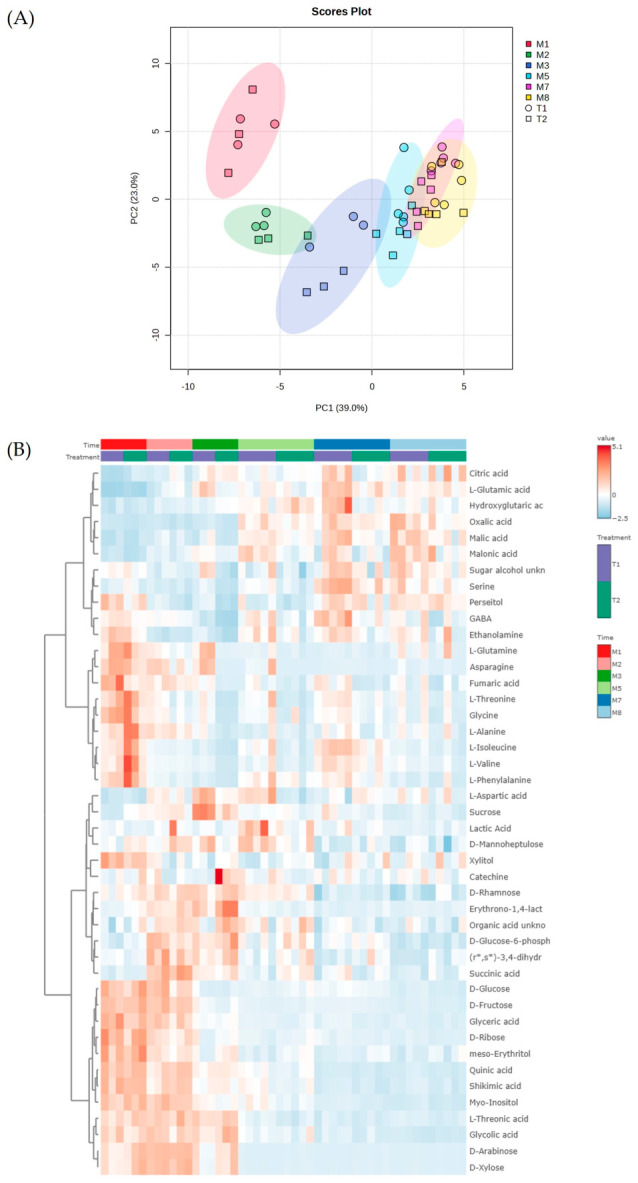
Polar metabolite profiling of the mesocarp of avocado fruit. (**A**) Principal component analysis (PCA) score-plot displaying the first two components and explained variance and (**B**) heatmap representation during different developmental stages (M1: February; M2: March; M3: May; M5: July; M7: September and M8: Harvest) and treatments (T1: 100% ETo and T2: 40% ETo). The columns represent biological replicates for each developmental stage and treatment, and the rows correspond to the metabolites. The similarity measure used to cluster the different features was based on Euclidean distance and Ward’s linkage from three or five biological replicates as detailed in the experimental design.

Principal component analysis (PCA; Figure 8A) of polar metabolites revealed separation of avocado fruit mainly by developmental stage, explaining 62.0% of the variance with the first two components. This indicates that polar metabolic profiles of the fruit underwent pronounced changes during development. Compared with the more advanced stages, the initial stages of development (M1: February and M2: March) exhibited a greater degree of separation. The polar metabolite profiles of samples collected in July (M5) and September (M7) were very similar to the profiles at harvest (M8). A two-way ANOVA analysis (FDR cutoff, 0.05; Supplementary Table S1) revealed the importance of the developmental stage (15 metabolites) and the shared effect of the developmental stage × irrigation treatment (15 metabolites) interaction, with a smaller contribution from interaction alone (1 metabolite). In fact, rather than acting independently, irrigation treatment effects are largely dependent on the developmental stage. Within each development stage, partial separation between the irrigation treatments was observed mainly along PC2 (23.0%). Heatmap analysis (Figure 8B) corroborated the PCA results and revealed groups of samples according to fruit development stage. Early stages were characterized by a relatively high abundance of organic acids, including malic, citric, and quinic acids. A greater abundance of amino acids, such as *L*-alanine, *L*-glutamine, and *L*-aspartic acid were also observed. Regarding the irrigation regime, there was a moderate response in metabolites related to osmotic adjustment, such as GABA and serine. Conversely, the later stages revealed an accumulation of sugars, primarily glucose, fructose, and sucrose, with only minor variations observed among the different irrigation treatments.

**Figure 9:**
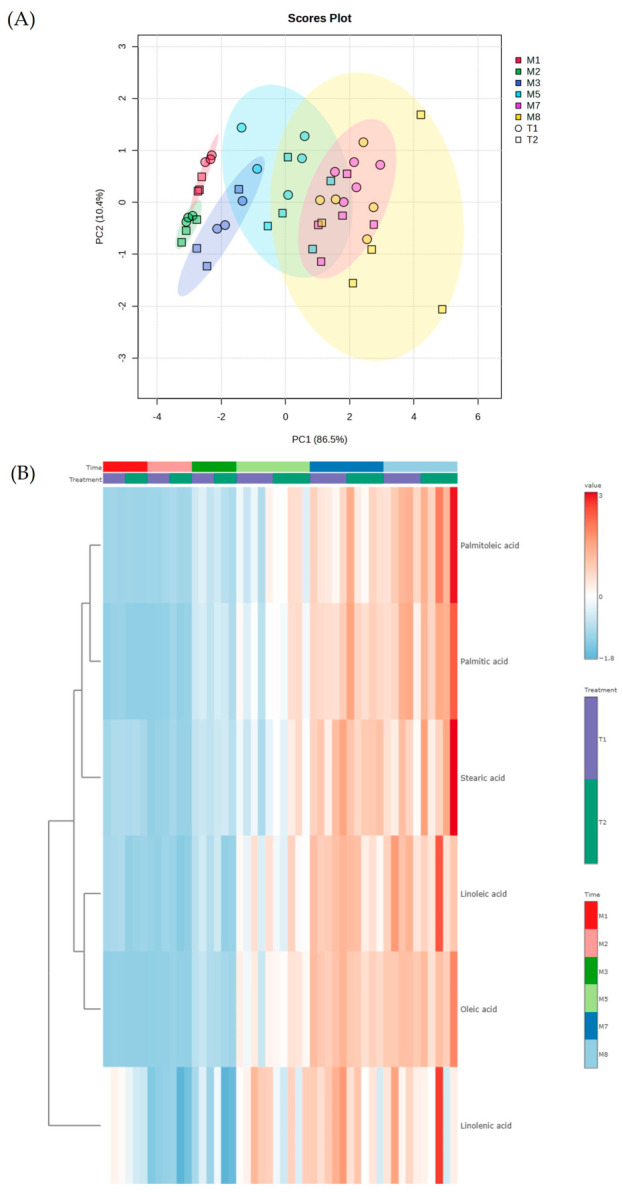
FAME profiles of the mesocarp of avocado fruit. (**A**) Principal component analysis (PCA) score plot displaying the first two components and explained variance and (**B**) heatmap representation during different developmental stages (M1: February; M2: March; M3: May; M5: July; M7: September and M8: Harvest) and treatments (T1: 100% ETo and T2: 40% ETo). The columns represent biological replicates for each developmental stage and treatment, and the rows correspond to the metabolites. The similarity measure used to cluster the different features was based on Euclidean distance and Ward’s linkage from three or five biological replicates as detailed in the experimental design.

Regarding the FAME profile, PCA separated the samples by stage of development, with PC1 explaining 86.5% of the variance and the first two components together explaining 96.5% of the variance (Figure 9A). The main fatty acids present in avocado are palmitoleic, palmitic, stearic, linoleic, linolenic, and oleic acids [19,20]. The heatmap (Figure 9B) shows that the levels of fatty acids such as palmitic (C16:0), stearic (C18:0), and oleic (C18:1) acids increased throughout fruit development. In contrast, the levels of polyunsaturated fatty acids (linoleic acid, C18:2, and linolenic acid, C18:3) decreased. There was no significant difference in the irrigation regime as also revealed by a two-way ANOVA (FDR cutoff, 0.05; Supplementary Table S2), suggesting that stress applied in the early stages of development does not affect the overall fatty acid composition.

**Figure 10:**
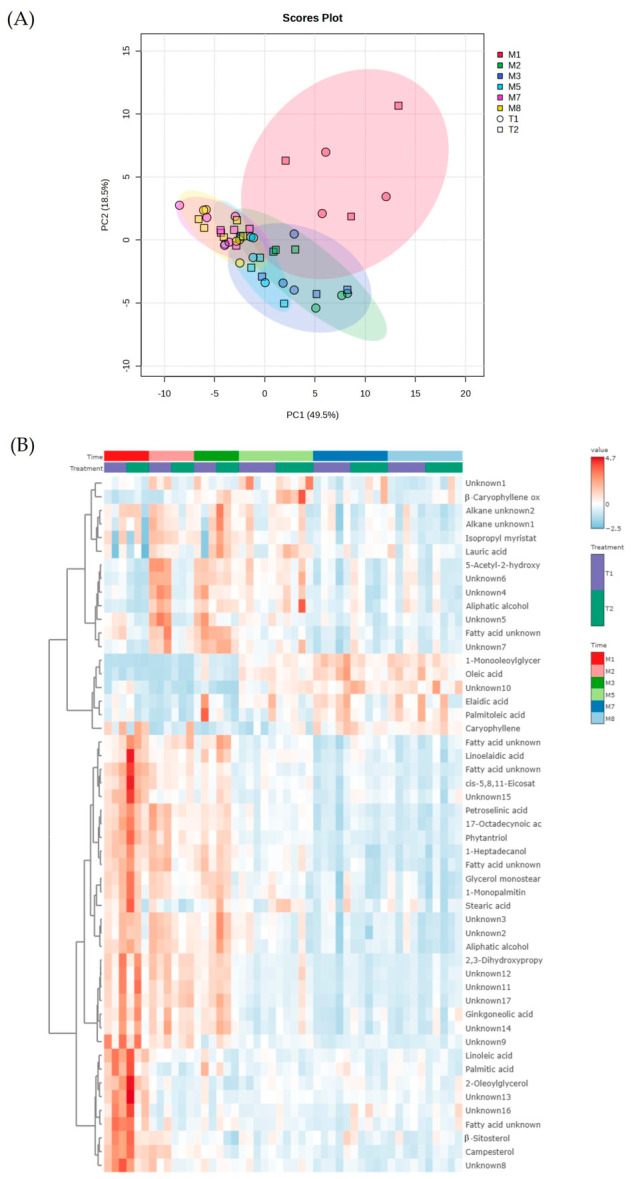
Nonpolar metabolite profiles of the mesocarp of avocado fruit. (**A**) Principal component analysis (PCA) score plot displaying the first two components and explained variance and (**B**) heatmap representation during different developmental stages (M1: February; M2: March; M3: May; M5: July; M7: September and M8: Harvest) and treatments (T1: 100% ETo and T2: 40% ETo). The columns represent biological replicates for each developmental stage and treatment and the rows correspond to the metabolites. The similarity measure used to cluster the different features was based on Euclidean distance and Ward’s linkage from three or five biological replicates as detailed in the experimental design.

Consistent with the trends observed for polar metabolites and FAMEs, nonpolar metabolites were also affected by fruit development. In the PCA (Figure 10A), component 1 separated the fruit according to stage of fruit development and explained 49.5% of the total variation, while component 2 accounted for 18.5% of the explained variation. The changes observed in lipophilic compounds, long-chain alcohols, and fatty acids were due to fruit development and showed a very low effect of the irrigation treatment, as also revealed by the two-way ANOVA (FDR cutoff, 0.05; Supplementary Table S3).

**Figure 11:**
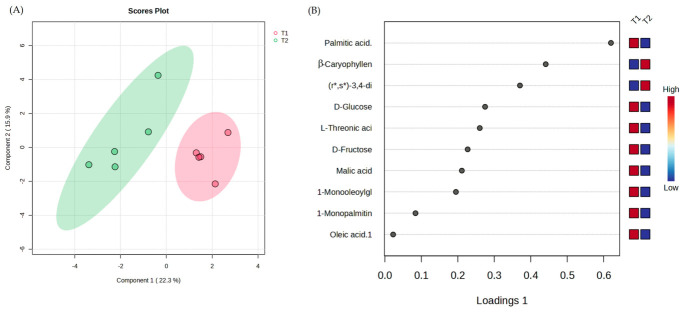
Nonpolar metabolite profiles of the mesocarp of avocado fruit. (**A**) Sparse partial least squares–discriminant analysis (sPLS-DA) score plot displaying the first two components and explained variance for mesocarp samples at harvest (M8) and two irrigation treatments (T1: 100% ETo and T2: 40% ETo). (**B**) Correlation loading plot for the first component with the most influential metabolites selected by the sparse PLS-DA. Each left row corresponds to the selected metabolites based on correlation loading contribution (*X*-axis).

Finally, the effect of the irrigation treatment was evaluated at harvest (M8) considering all metabolites (polar, FAMEs, and nonpolar metabolites) using a sparse partial least square discriminant selection (sPLS-DA; Figure 11) and a T-test (FDR cutoff, 0.05). The sPLS-DA analysis resulted in treatments being separated mainly by factor 1 (22.3% of the variance explained; Figure 11A). Considering the FDR value of 0.05, the T-test revealed no metabolite to be differentially expressed. The correlation loadings of the metabolites selected via sPLS-DA in the first component (Figure 11B) corresponded to palmitic acid, *β*-Caryophyllene oxide, (r*, s*)-3,4-dihydroxybutanoic acid, *D*-Glucose, *L*-threonic acid, *D*-fructose, Malic acid, 1-Monooleoylglycerol, 1-Monopalmitin, and oleic acid. All were present at a higher relative abundance in the control treatment (T1 = 100% ETo) except for *β*-Caryophyllene oxide and (r*, s*)-3,4-dihydroxybutanoic acid, which were present in higher amounts in T2 (40% ETo). Pathway enrichment analysis results (Supplementary Table S7) should be interpreted with caution since all pathways displaying a −log(*p*) > 1.3 had an FDR > 0.05.

## 3. Discussion

In this study, a differential water status in plants was successfully generated by applying different irrigation regimes during a critical development period, as evidenced by changes in the soil volumetric water content (VWC) and stem water potential (SWP). Deficit and excess irrigation induced physiological stress, which is consistent with previously reported findings for avocado trees growing under Mediterranean conditions [17,21]. The decrease in SWP in the deficit irrigation treatments confirms that water restriction directly affects plant water status. The 120% ETo treatment also resulted in suboptimal SWP and reduced FPSII, indicating that excess water reduces root oxygen availability and, consequently, alters photosynthetic performance. This sensitivity to water deficit and excess is consistent with the depth of the root system and low tolerance to hypoxic conditions, which are characteristics of avocado trees [4,22,23]. The absence of significant effects on F_V_/F_M_ suggests that the stress imposed was not sufficient to cause damage to PSII. These results emphasize that avocado trees function within a limited optimal range of water availability and that excess and deficit conditions lead to measurable physiological stress.

Based on these results, the leaves and fruits were characterized. Deficit and excess irrigation induced physiological stress, but neither affected the overall nutritional nor the chlorophyll content of the leaves. Also, no differences in stomatal number were observed. This behavior might indicate that the imposed water deficit did not trigger structural or biochemical acclimation responses in avocado leaves. In *Persea americana*, the mineral content of leaves is tightly regulated and largely dependent on the developmental stage of the leaves rather than on short-term environmental fluctuations [14]. Similarly, stomatal density and anatomy are determined during leaf expansion and remain relatively insensitive to moderate water deficits imposed after full leaf development, with drought responses occurring mainly through functional regulation of stomatal conductance [24,25,26]. The maintenance of chlorophylls content also suggests that the photosynthetic apparatus was not structurally affected, supporting the idea that water stress does not affect chlorophyll metabolism (synthesis and degradation) [11,27].

Water availability strongly influenced avocado fruit growth dynamics and associated quality-related attributes. Deficient irrigation significantly reduced fruit size and final weight, which is consistent with decreased cell expansion due to reduced turgor pressure and limited water-driven growth. In contrast, 120% ETo increased fruit size, likely by prolonging the period of cell expansion and increasing fruit water content, rather than by stimulating structural dry matter accumulation. These responses are consistent with the observations of previous studies demonstrating a close coupling between water supply and fruit growth in avocado [28]. Despite reduced fruit size under deficit irrigation, DM content increased, particularly under the 40% ETo treatment. A concentration effect best explains this increase in DM, due to reduced fresh weight accumulation rather than enhanced dry-matter accumulation. In avocado, water limitation commonly reduces fruit water content to a greater extent than carbon accumulation, leading to higher DM content in smaller fruit and an apparent advancement in physiological maturity [29]. Accordingly, the observed increase in DM under deficit irrigation likely reflects altered fruit–water relationships and developmental timing rather than fundamental changes in carbon partitioning [7,30]. Water availability also influenced the fruit’s external appearance. Reduced irrigation (40% ETo) resulted in smaller fruits with a higher DM content and a more yellowish-green color.

In contrast, 120% ETo resulted in larger fruits with a darker green color, which is consistent with delayed maturation under excess water availability. Taken together, these findings suggest that preharvest water management primarily affects avocado fruit growth by influencing cell expansion and water accumulation. Associated changes in the DM content and skin color reflect shifts in the water balance. This information contributes to our understanding of how preharvest water availability may subsequently influence postharvest fruit quality.

By the end of the irrigation regime, calcium levels decreased, with the lowest levels observed in the 40% ETo treatment. This result is consistent with the literature: calcium accumulation is sensitive to reduced xylem flow during the early stages of fruit development [12,13,31]. Since Ca does not move through the phloem, any limitation in xylem flow decreases the accumulation of this nutrient [32,33]. However, when irrigation was re-established, calcium levels were similar across all treatments and remained within the expected range until harvest. These observations suggest that, following the restoration of soil water status, fruit transpiration rates increased, thereby enhancing Ca influx and resulting in higher Ca concentrations within the fruit tissues. Similar recovery responses have been described in other perennial fruit crops subjected to transient water deficit [29]; thus, the timing of abiotic stress is extremely important.

The physiological and quality parameters were measured during cold storage. In this study, the ethylene and respiratory rates were low for all treatments. This behavior is similar to that described by other authors for the ’Hass’ cultivar stored under cold conditions, where low temperatures reduce metabolic activity and delay the climacteric peak associated with ripening [34,35,36]. Similar responses have been described in several avocado cultivars such as ’Fuerte’, ’Edranol’, and ’Ettinger’, where ethylene synthesis and respiratory metabolism remain suppressed during cold storage but rapidly increase when fruit are transferred to shelf-life conditions [19,37,38]. However, no differences were observed between treatments, suggesting that the irrigation regimes in this study had limited influence on the fruit’s postharvest ripening physiology.

Regarding the softening rate, all treatments exhibited similar behavior. Firmness loss during cold storage and shelf life followed the typical softening pattern associated with avocado ripening, involving cell wall degradation and changes in pectin structure mediated by cell-wall-modifying enzymes [39]. Similar softening kinetics have been widely described in avocado fruit during postharvest ripening [19,34]. The comparable softening rates across treatments suggest that the irrigation regimes applied during fruit development did not substantially affect the fruit’s ripening capacity.

Metabolomic analysis revealed that the fruit developmental stage was the main factor shaping the metabolic composition of avocado mesocarp. In contrast, the early-season water deficit imposed in this study induced only minor metabolic adjustments. Polar metabolites, fatty acids, and nonpolar compounds consistently showed clear temporal shifts associated with fruit growth and maturation. Early developmental stages were characterized by higher levels of organic acids and amino acids, reflecting intense metabolic activity associated with cell division and expansion [6,40]. As development progressed, the metabolic profile shifted toward increased accumulation of sugars and lipids, which are essential components of avocado fruit quality and energy storage [6,19]. The increase in saturated and monounsaturated fatty acids, such as palmitic, stearic, and oleic acids, during fruit growth is consistent with the accumulation of lipids in the avocado mesocarp. Avocado accumulates triglycerides during development, with oleic acid accounting for their majority at maturity [6,19]. In contrast, the limited effect of the irrigation treatment on most metabolite classes suggests that moderate water deficit applied during early fruit development does not substantially disrupt the metabolic pathways associated with carbon partitioning and lipid biosynthesis.

In response to a water deficit, a subtle change in metabolites associated with osmotic adjustment is observed, including GABA and serine. GABA accumulation has also been observed in response to other types of stress, where this metabolite contributes to cellular osmotic regulation and stress signaling. Increased GABA levels have been observed in several fruit species under water-deficit conditions, suggesting that this pathway plays an important role in metabolic acclimation to stress [41].

Our results suggest that developmental processes regulate avocado fruit metabolism and can maintain relative metabolic stability under moderate water-deficit conditions. Nevertheless, the metabolic adjustments observed during fruit growth may modulate fruit biochemical status at harvest and, in turn, postharvest performance and quality. Metabolites selected at harvest via sPLS-DA represent a mixture of primary metabolism (central carbon metabolism, organic acids, sugars) and lipid-related or secondary metabolites, suggesting coordinated shifts in energy metabolism, membrane dynamics, and stress-related pathways. These metabolites were mostly present in higher relative abundance in the 100% ETo treatment than in the water deficit treatment (40% ETo) except for β-Caryophyllene oxide and (r*, s*)-3,4-dihydroxybutanoic acid, which were higher under 40% ETo. These last metabolites have been previously reported as defense-related compounds and involved in osmotic regulation [42,43].

## 4. Materials and Methods

This study was conducted in a commercial orchard located in Cabildo (Chile; 32°24′46″ S 70°54′26″ W) during two consecutive growing seasons (2022/2023 and 2023/2024). Six-year-old trees (*Persea americana Mill*. cv. ‘Hass’ grafted onto ‘Mexicola’) were spaced 5 m between rows and 2 m between trees. The orchard had a temperate Mediterranean-type climate, characterized by dry and warm summers. In this orchard, the flowering period lasted up to three months (September–November), and fruit set occurred immediately afterward (December–January), followed by a phase of intensive fruit growth. The fruit could remain on the tree for 400 days before harvest, which can begin as early as September and extend for several months.

### 4.1. Irrigation Regimes Treatments

Different irrigation regimes were applied based on the reference evapotranspiration (ETo). The treatments were as follows: T1 (control) = irrigation with amounts of water equal to 100% ETo; T2 = irrigation with amounts of water equal to 40% ETo; T3 = irrigation with amounts of water equal to 60% ETo; T4 = irrigation with amounts of water equal to 120% ETo. For each treatment, a row with similar characteristics was selected. The irrigation system was replaced for 10 trees in this row. The conventional microjet irrigation system was replaced with a drip system with different flow rates and spacings to allow the application of these irrigation regimes. The treatments were applied from January to March, after which conventional orchard irrigation was reinstated. The physiological variables of three trees per treatment were monitored periodically. Harvest was carried out at a fruit dry matter content (DM) of 24–26%. Environmental conditions were continuously monitored throughout the experimental period using HOBO sensors (Onset Computer Corporation, Bourne, MA, USA) and PAR sensors (Apogee instruments INC, Logan, UT, USA) installed within the orchard. Thus, air temperature, relative humidity, and solar radiation were recorded at hourly intervals.

### 4.2. Soil and Plant Water Status

Soil and plant water status were evaluated in parallel throughout the experimental period. Volumetric water content (VWC) was continuously monitored using TEROS-10 sensors (METER Group Inc., Pullman, WA, USA) connected to ZL6 data loggers installed at a soil depth of 30–60 cm between two emitters and at 50 cm from the trunk of a central tree per treatment. In addition, the VWC was measured twice per month at a depth of 30 cm for three trees per treatment. Measurements were taken at the four cardinal points within a small excavation using a portable TEROS-10 sensor (METER Group Inc., Pullman, WA, USA). Stem water potential (SWP) was measured twice per month at midday using a Scholander-type pressure chamber on healthy, fully expanded mature leaves (nine leaves per treatment), which were covered with plastic bags and aluminum foil for at least 30 min to equilibrate SWP. The results are expressed in MPa.

### 4.3. Photosynthetic Efficiency

Photosynthetic efficiency was evaluated using a portable chlorophyll fluorescence analyzer (Photosynthesis Yield Analyzer Mini-PAM II, Walz, Germany). Measurements were performed on young, fully expanded, non-senescent leaves from the middle third of the canopy tree (nine leaves per treatment). The maximum quantum efficiency of PSII (F_V_/F_M_) was determined after dark adaptation for at least 30 min using leaf clips, followed by the application of a saturating light pulse to obtain the minimum (F_0_) and maximum (F_M_) fluorescence, from which the variable fluorescence (F_V_ = F_M_ − F_0_) and the F_V_/F_M_ ratio were calculated. Minimum fluorescence (F_0_) corresponds to the fluorescence emitted when all PSII reaction centers are open. Maximum fluorescence (F_M_) is obtained after a saturating light pulse when all photosystem II (PSII) reaction centers are closed. Variable fluorescence (F_V_ = F_M_ − F_0_) represents the variable component of fluorescence. The F_V_/F_M_ ratio is used as an estimate of the maximum quantum efficiency of PSII photochemistry under dark-adapted conditions. The effective quantum efficiency of PSII (ΦPSII or Y(II)) was measured in light-adapted leaves under ambient light conditions, without prior dark adaptation, between 10:00 and 13:30 h. Leaf temperature was simultaneously recorded with the integrated thermocouple in the Mini-PAM II and served as an indicator of plant thermal and water status.

### 4.4. Leaf Nutrient Content

The mineral nutrient composition was determined in leaf samples from the spring flush for each treatment, collected prior to autumn sprouting. Eight to ten fully expanded leaves from three different trees were picked (per treatment), washed in a 0.1% Tween 20 and 0.1 M HCl solution, and rinsed in deionized water. Afterward, the samples were oven-dried at 75 °C for 72 h and ground into powder. Nutrient concentration (N, P, K, Ca, Mg, Cu, Fe, Mn, Zn, Na and B) was determined using an MP-AES 4200 microwave plasma atomic emission spectroscope (Agilent Technologies, Santa Clara, CA, USA) after acid digestion with nitric acid and hydrogen peroxide at 180 °C for 20 min in a microwave (Anton Paar 3200, Graz, Austria) [44].

### 4.5. Stomatal Density

Stomatal density was determined for three mature leaves per treatment. Stomatal imprints in attached leaves (5 zone per leaf) were made by applying nail varnish on the abaxial and adaxial surfaces of the leaves, avoiding the midrib and the leaf margin. The stomatal imprints were analyzed with a light microscope (Biopstik Technology, Miaoli, Taiwan). Stomatal density (n°/mm^2^) was determined by counting all stomata in the image and extrapolating to 1 mm^2^, as described by Opazo et al. [45].

### 4.6. Chlorophyll Content

Chlorophylls were extracted from 50 mg of lyophilized leaves or exocarp using the method reported by Miazek and Ledakowicz [46], with some modifications. Methanol was used as a solvent and extracting agent to measure absorbance at 665, 652, and 470 nm using a microplate spectrophotometer (EpochTM, Biotek, Winooski, VT, USA). The final content was calculated following the equations proposed by Lichtenthaler [47].

(1) $$Total\;Chlorophyll\left(\mu g\;{mL}^{-1}\right)=\left(1.44\times A_{665}\right)+\left(24.93\times A_{652}\right)$$

(2) $$Chlorophyll\;a\left(\mu g\;{mL}^{-1}\right)=\left(16.72\times A_{665}\right)-\left(9.16\times A_{652}\right)$$

(3) $$Chlorophyll\;b\left(\mu g\;{mL}^{-1}\right)=\left(34.09\times A_{652}\right)-\left(15.28\times A_{665}\right)$$

(4) $$Total\;Carotenoids\left(\mu g\;{mL}^{-1}\right)=\left[\left(1000\times 470\right)-\left(1.63\times C_{a}\right)-\left(104.9\times C_{b}\right)\right]\times 221^{-1}$$

The results were corrected using the concentration of the samples (g mL^−1^), obtaining a final concentration of pigments expressed as [mg kg^− 1^ FW sample].

### 4.7. Fruit Size, Dry Matter (DM), and Fruit Nutrient Content

Each month, ten fruits per treatment were characterized by weight (g), as well as by their equatorial and polar diameter (mm). Additionally, the DM content was determined by oven-drying mesocarp or epicarp samples at 103 °C until constant weight was reached. The dry matter content was expressed as a percentage (g of dry matter per 100 g of epicarp or mesocarp). The nutrient contents in the fruits were measured in a similar manner to that described for the leaves (Section 4.4). Three to five fruits were collected from three different trees for each treatment. The skin was separated from the pulp and oven-dried.

### 4.8. Harvest and Postharvest Quality

For each harvest (DM content ranged from 24 to 26%), 150 fruits were collected per treatment, and 30 were immediately evaluated. The remaining avocados (*n* = 120) were packaged in three ventilated plastic boxes (*n* = 40 avocados/box) and stored under air atmosphere at 5 ± 0.5 °C with a relative humidity of 80–85% for 30 days. Evaluations were performed at 10-day intervals (10, 20, and 30 d; *n* = 15 fruits per treatment). By the end of cold storage (30 d), the fruit samples (*n* = 75) were maintained at shelf-life temperature (20 ± 0.5 °C and 45% RH) until they reached the ready-to-eat time (RTE) stage (mesocarp firmness < 14 N).

#### Physiological and Global Quality Parameters

Physiological and quality parameters were measured individually on 15 avocado fruit per treatment within each evaluation time, i.e., at harvest, every 10 days during storage, and during shelf life. For the measurement of the respiration and ethylene production rates, a single avocado was placed in a 1.6 L polyethylene (not ethylene release material) container and sealed hermetically for 2–4 h, thus avoiding excessive accumulation of carbon dioxide. For the respiration rate, CO_2_ production was measured by injecting 1 mL of gas, taken from the container’s headspace, into a gas analyzer (PBI-Dansensor Checkmate 9900, Ringsted, Denmark), and values were expressed as mL CO_2_ kg^−1^ h^−1^. For the measurement of ethylene, 1 mL of gas was taken from the headspace and injected into a gas chromatograph (Shimadzu GC 8A, Tokyo, Japan) equipped with an alumina column (Supelco (Bellefonte, PA, USA) 80/100 Porapak custom column with dimensions 75 cm × 5 mm × 3 mm) and a flame ionization detector (FID). The oven and injector temperatures were 40 and 150 °C, respectively. Values were expressed as μL C_2_H_4_ kg^−1^h^−1^ [48]. The firmness of each fruit was evaluated using a non-destructive texture analyzer (Model TA.XT plus C, Stable Micro Systems Ltd., Godalming, Surrey, UK) fitted with a 10 mm diameter cylinder probe (Ø), 0.5 N trigger threshold, and 8 mms^−1^ measurement speed. The compression force was recorded in Newton (N) at a deformation of 2 mm. The firmness was determined at two equidistant points on the equatorial region of each whole fruit [49]. Skin color development was assessed visually using a hedonic scale (from 1 to 5), where 1 = 100% of the skin surface is green, 2 = 20% of the skin surface is colored black/purple (violet), 3 = 60% of the skin surface is colored black/purple (violet), 4 = 100% of the skin surface is purple (violet), and 5 = 100% of the skin surface is black. The skin color quality was evaluated with a reflectance colorimeter (CR-300; Minolta, Tokyo, Japan), using the *CIELab* color system. Two equidistant color measurements were performed around the equator of each fruit. Physiological disorders such as browning and lenticel damage were visually assessed using a hedonic scale (1 to 4), where 1 = no occurrence, 2 = slight, 3 = moderate, 4 = severe.

### 4.9. Metabolomics

Metabolomic analyses were performed on mesocarp and epicarp tissues using complementary gas chromatography-based platforms to determine fatty acids and polar and nonpolar metabolites.

#### 4.9.1. GC-FID Fatty Acid Analysis

Fatty acids were determined in mesocarp and epicarp tissues following the protocol described by Campos et al. [50] and using the methylation procedure reported by Meurens et al. [51]. Fatty acid methyl esters (FAMEs) were determined using a GC-FID (Agilent Technologies, Santa Clara, CA, USA), under the chromatographic conditions described by Olmedo et al. [19]. FAMEs were identified and quantified through comparison with external standards, and the results were expressed as g fatty acid kg^−1^ DW.

#### 4.9.2. GC-MS Analysis of Polar Metabolites

GC-MS polar metabolite analyses were carried out on mesocarp and epicarp tissues; polar metabolites were extracted and derivatized according to a method described by Fuentealba et al. [52]. Chromatographic peaks were assigned and identified by comparing retention times and mass spectra to a custom-built library of commercial standards and the NIST14 library using Mass Hunter Quantitative 10.0 software (Agilent Technologies, Santa Clara, CA, USA). Metabolites were reported based on relative quantification, using phenyl β-D-glucopyranoside as an internal standard, sample dry weight, and a pooled quality control (QC) sample representative of all analyzed samples.

#### 4.9.3. GC-MS Nonpolar Metabolite Analysis

The nonpolar metabolites were extracted and derivatized according to protocol [53]. Samples were analyzed using an Agilent 7890B gas chromatograph coupled to a 5977A single-quadrupole mass spectrometer equipped with an electron impact ionization source (Agilent Technologies, Santa Clara, CA, USA). Metabolites were separated and detected under chromatographic and mass spectrometric conditions [54]. Metabolites were identified using Mass Hunter Quantitative software (Agilent Technologies, Santa Clara, CA, USA) and the NIST14 mass spectral library (NIST, USA) and were reported as relative abundances.

### 4.10. Experimental Design and Statistical Analysis

The experiments were conducted according to a completely randomized design. Before analyzing percentages, the data were arcsine-transformed, with the non-transformed values used for presentation in the figures. The data were subjected to a statistical analysis of variance (ANOVA), and the means were separated using Tukey’s test at 5% significance using the statistical software InfoStat (Version 2015, Universidad Nacional de Córdoba, Córdoba, Argentina). Data normalization consisted of mean-centering and dividing by the standard deviation of each variable. Principal component analysis (PCA) and heat map analysis were performed on the normalized dataset obtained by GC-MS. Heatmaps were built using the similarity measure to cluster the different variables based on Euclidean distance and Ward’s linkage. Additionally, a two-way ANOVA was applied to metabolomics data using a false discovery rate adjusted to 0.05, considering the developmental stage and water restriction treatments as factors. Three (M1, M2, M3, and M5) and five (M7 and M8) biological replicates were used. Sparse partial least squares–discriminant analysis (PLS-DA) and pathway analysis were carried out on mesocarp samples only at harvest, comparing T1 = 100% ETo and T2 = 40% ETo using five biological replicates (*n* = 5) and the *Arabidopsis thaliana* pathway library. These analyses were carried out in MetaboAnalyst 6.0 software (Meta, Edmonton, AB, Canada).

## 5. Conclusions

Water restriction (40% and 60% ETo) and overirrigation (120% ETo) affect the physiological status, as evidenced by decreases in stem water potential and alterations in the effective quantum efficiency of PSII (ΦPSII). However, this temporary stress does not alter long-term structural or biochemical parameters, with the nutritional balance of the leaves.

Water availability strongly determines the fruit’s physical characteristics and maturity. Deficient irrigation restricts cell expansion, significantly reducing the fruit’s final weight and size. This generates a concentration effect that increases dry matter levels and promotes a green-yellowish coloration, simulating an advance in physiological maturity. Conversely, excessive irrigation promotes a larger size and darker green coloration, diluting the DM content and delaying ripening.

The metabolite profiles (polar metabolites, nonpolar metabolites, and fatty acid methyl esters) in the mesocarp are determined mainly based on the ontogenetic (developmental) state of the fruit rather than early-irrigation conditions. The metabolites most strongly contributing to the discrimination at harvest, as revealed by sPLS-DA, comprised primary metabolites (central carbon metabolism intermediates, organic acids, and sugars) and lipid-related or secondary compounds. This pattern indicates coordinated adjustments in energy metabolism, membrane dynamics, and stress-responsive pathways. Notably, most of these metabolites were present at lower abundance in the 40% ETo treatment, except for β-caryophyllene oxide and (r*, s*)-3,4-dihydroxybutanoic acid, which exhibited higher levels.

Regarding the consumption quality, fruits subjected to 120% ETo showed the greatest vulnerability to physiological disorders, evidencing a higher incidence of vascular browning during their shelf life.

## Figures and Tables

**Table 1:** Mineral content in leaves.

Mineral	Optimal Range *	100% ETo	40% ETo	60% ETo	120% ETo
Nitrogen (N; %)	[2.0–2.5]	2.3 ± 0.20 ^a^	2.3 ± 0.20 ^a^	2.3 ± 0.20 ^a^	2.2 ± 0.10 ^a^
Phosphorus (P; %)	[0.1–0.2]	0.1 ± 0.01 ^a^	0.2 ± 0.01 ^a^	0.2 ± 0.02 ^a^	0.2 ± 0.01 ^a^
Potassium (K; %)	[0.8–2.0]	1.6 ± 0.10 ^bc^	1.7 ± 0.10 ^c^	1.5 ± 0.10 ^ab^	1.3 ± 0.10 ^a^
Calcium (Ca; %)	[1.0–2.0]	2.0 ± 0.10 ^a^	1.9 ± 0.10 ^a^	1.9 ± 0.20 ^a^	2.2 ± 0.10 ^b^
Magnesium (Mg; %)	[0.4–1.0]	0.5 ± 0.03 ^a^	0.5 ± 0.04 ^a^	0.5 ± 0.03 ^a^	0.5 ± 0.02 ^a^
Iron (Fe; mg kg^−1^)	[50–900]	137.0 ± 8.90 ^b^	137.1 ± 7.90 ^b^	117.2 ± 7.60 ^a^	111.0 ± 7.20 ^a^
Zinc (Zn; mg kg^−1^)	[30–200]	71.1 ± 4.60 ^d^	46.2 ± 3.00 ^b^	54.0 ± 3.50 ^c^	35.1 ± 2.30 ^a^
Sodium (Na; mg kg^−1^)	[<2500]	59.2 ± 3.80 ^b^	73.1 ± 4.80 ^c^	48.1 ± 3.10 ^a^	42.2 ± 2.70 ^a^
Boron (B; mg kg^−1^)	[30–90]	60.1 ± 3.90 ^a^	71.1 ± 4.60 ^b^	53.1 ± 3.50 ^a^	67.2 ± 4.40 ^a^
Manganese (Mn; mg kg^−1^)	[50–700]	200.1 ± 13.00 ^b^	174.1 ± 11.30 ^a^	165.0 ± 10.10 ^a^	199.1 ± 13.00 ^a^

* The optimal range column was obtained from González-Vences et al. [18]. Data represent the means ± SD (n = 9). Different letters indicate significant differences among treatments according to Tukey’s test (*p* < 0.05).

**Table 2:** Quality variables at harvest for fruits from different irrigation regimes.

Treatment	DM (%)	Weight (g)	*hue Angle*	Firmness (N)	Ethylene (μL C_2_H_4_ kg^−1^h^−1^)	CO_2_ (mL CO_2_ kg^−1^h^−1^)
100% ETo	25.2 ± 0.5 ^a^	160.3 ± 5.7 ^bc^	124.9 ± 1.5 ^b^	94.4 ± 7.6 ^a^	0.18 ± 0.04 ^a^	45.6 ± 3.4 ^a^
40% ETo	26.1 ± 0.3 ^a^	125.3 ± 4.7 ^a^	122.6 ± 2.1 ^a^	96.6 ± 12.1 ^a^	0.21 ± 0.04 ^a^	45.2 ± 5.7 ^a^
60% ETo	24.9 ± 0.2 ^a^	150.7 ± 4.7 ^ab^	124.7 ± 1.8 ^b^	97.8 ± 9.6 ^a^	0.22 ± 0.06 ^a^	40.0 ± 3.1 ^a^
120% ETo	25.3 ± 0.4 ^a^	183.6 ± 5.4 ^c^	124.6 ± 1.9 ^b^	97.2 ± 10.8 ^a^	0.12 ± 0.02 ^a^	44.3 ± 1.5 ^a^

Data represent the means ± SD (*n* = 15). Different letters indicate significant differences among treatments according to Tukey’s test (*p* < 0.05).

## Data Availability

The original contributions presented in this study are included in the article/Supplementary Materials. Further inquiries can be directed to the corresponding author.

## Supplementary Materials

The following supporting information can be downloaded at: https://www.mdpi.com/article/10.3390/plants15121807/s1, Figure S1: Stomatal density and chlorophylls content in leaf. (A) Stomatal density. (B) total chlorophyll. Bars represent the means of treatments ± SD. Different letters indicate significant differences among treatments (*p* < 0.05). Figure S2: Polar metabolite profiling of the exocarp of avocado fruit. (A) Principal component analysis (PCA) Score-plot displaying the first two components and explained variance and (B) heatmap representation during different developmental stages (M1: February; M2: March; M3: May; M5: July; M7: September and M8: Harvest) and treatments (T1: 100% ETo and T2: 40% ETo). The columns represent biological replicates for each developmental stage and treatment, and the rows correspond to the metabolites. The similarity measure was used to cluster the different features was based on Euclidean distance and Ward’s linkage from three or five biological replicates as detailed in the experimental design. Figure S3: FAME profiles of the exocarp of avocado fruit. (A) Principal component analysis (PCA) Score plot displaying the first two components and explained variance and (B) heatmap representation during different developmental stages (M1: February; M2: March; M3: May; M5: July; M7: September and M8: Harvest) and treatments (T1: 100% ETo and T2: 40% ETo). The columns represent biological replicates for each developmental stage and treatment, and the rows correspond to the metabolites. The similarity measure was used to cluster the different features was based on Euclidean distance and Ward’s linkage from three or five biological replicates as detailed in the experimental design. Figure S4: Nonpolar metabolite profiles of the exocarp of avocado fruit. (A) Principal component analysis (PCA) Score plot displaying the first two components and explained variance and (B) heatmap representation during different developmental stages (M1: February; M2: March; M3: May; M5: July; M7: September and M8: Harvest) and treatments (T1: 100% ETo and T2: 40% ETo). The columns represent biological replicates for each developmental stage and treatment and the rows correspond to the metabolites. The similarity measure was used to cluster the different features was based on Euclidean distance and Ward’s linkage from three or five biological replicates as detailed in the experimental design. Table S1. Two-way ANOVA polar metabolites mesocarp. Table S2. Two-way ANOVA FAMEs mesocarp. Table S3. Two-way ANOVA non polar metabolites mesocarp. Table S4. Two-way ANOVA polar metabolites exocarp. Table S5. Two-way ANOVA FAMEs exocarp. Table S6. Two-way ANOVA non-polar metabolites exocarp. Table S7. Results from pathway analysis against the *Arabidopsis thaliana* pathway library. Total: is the total number of compounds in the pathway; Hits: is the actually matched number from the user uploaded data; Raw *p* is the original *p* value calculated from the enrichment analysis; Holm *p* is the *p* value adjusted by Holm-Bonferroni method; FDR *p* is the *p* value adjusted using False Discovery Rate; Impact is the pathway impact value calculated from pathway topology analysis.

## Abbreviations

The following abbreviations are used in this manuscript:

FV/FM	Maximum quantum yield of photosystem II
DM	Dry Matter
Ca	Calcium
PAR	Photosynthetically Active Radiation
Tmax	Maximum air temperature
Tmin	Minimum air temperature
RH	Relative Humidity
ETo	Reference Evotranspiration
RTE	Ready Eat Time
PCA	Principal Component Analysis
FAMEs	Fatty Acid Methyl Esters
GC-MS	Gas Chromatography-Mass Spectrometry
GC-FID	Gas Chromatography-Flame Ionization Detector
QC	Quality Control
ANOVA	Statistical analysis of variance
RA	Regular Atmosphere
SWP	Stem Water Potential
SWP	Volumetric Water Content
FPSII	Effective quantum yield of the photosystem
