# Supplementary material for: Effect of Water Regimen on Fruit Growth, Metabolomic Profile, and Postharvest Quality of ‘Hass’ Avocados
Source: Plants (Basel). 2026 Jun 11;15(12):1807. doi: 10.3390/plants15121807 (PMC13306940; doi:10.3390/plants15121807)
Supplement: Supplementary file 1 [file plants-15-01807-s001.zip › Supplementary Figures.pdf]

Supplementary Materials

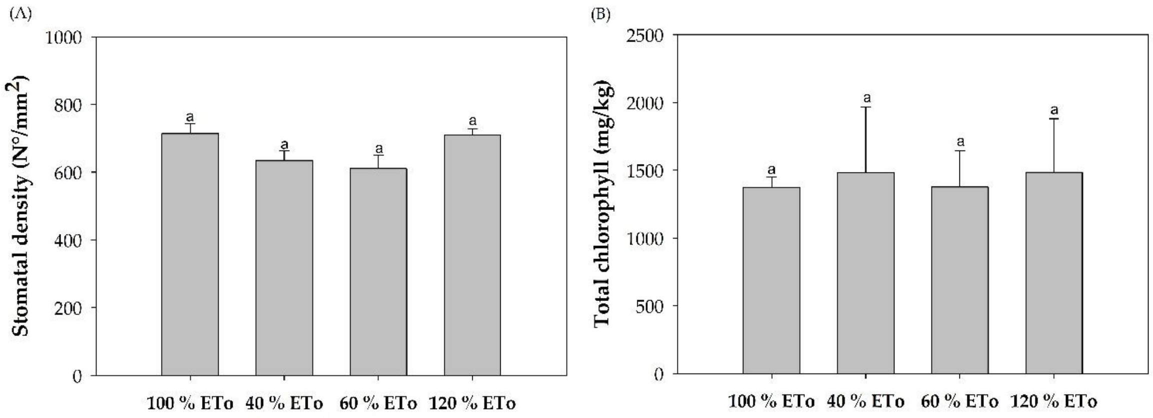

Figure S1. Stomatal density and chlorophylls content in leaf. A) Stomatal density. B) Total chlorophyll. Bars represent the means of treatments ± SD. Different letters indicate significant differences among treatments ($p < 0.05$).

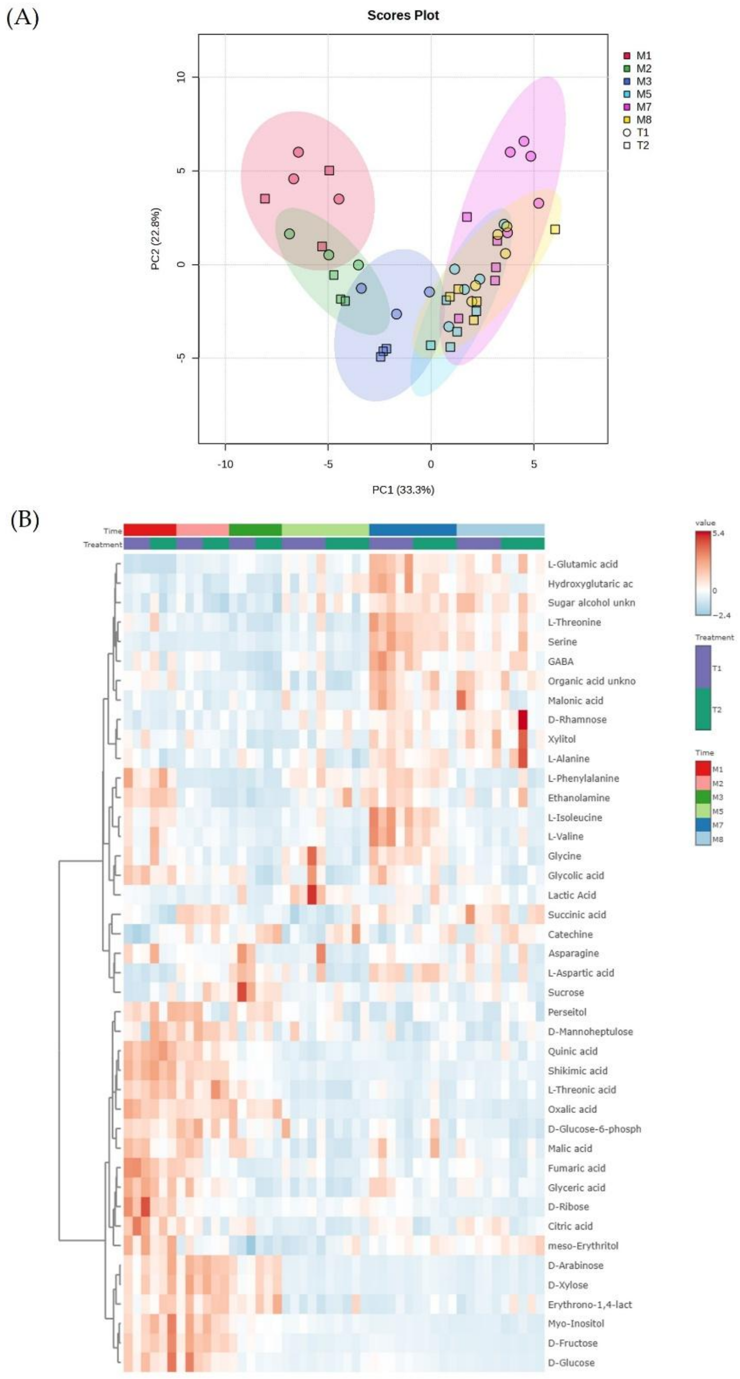

Figure S2. Polar metabolite profiling of the exocarp of avocado fruit. A) Principal Component Analysis (PCA) Score-plot displaying the first two components and explained variance and B) Heatmap representation during different developmental stages (M1: February; M2: March; M3: May; M5: July; M7: September and M8: Harvest) and treatments (T1: 100% ETo and T2: 40% ETo). The columns represent biological replicates for each developmental stage and treatment, and the rows correspond to the metabolites. The similarity measure was used to cluster the different features was based on Euclidean distance and Ward's linkage from three or five biological replicates as detailed in the experimental design.

(A)

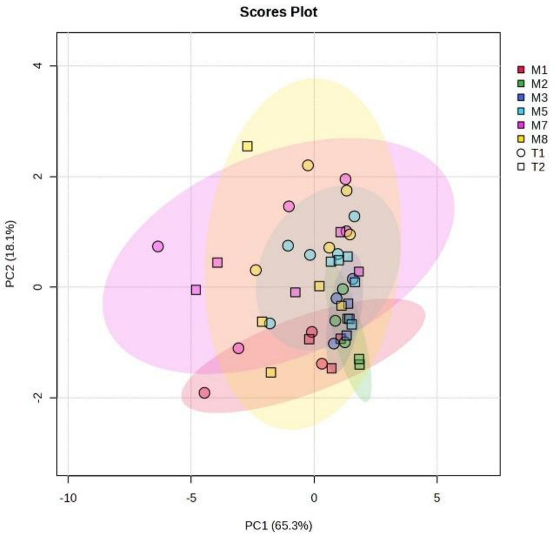

(B)

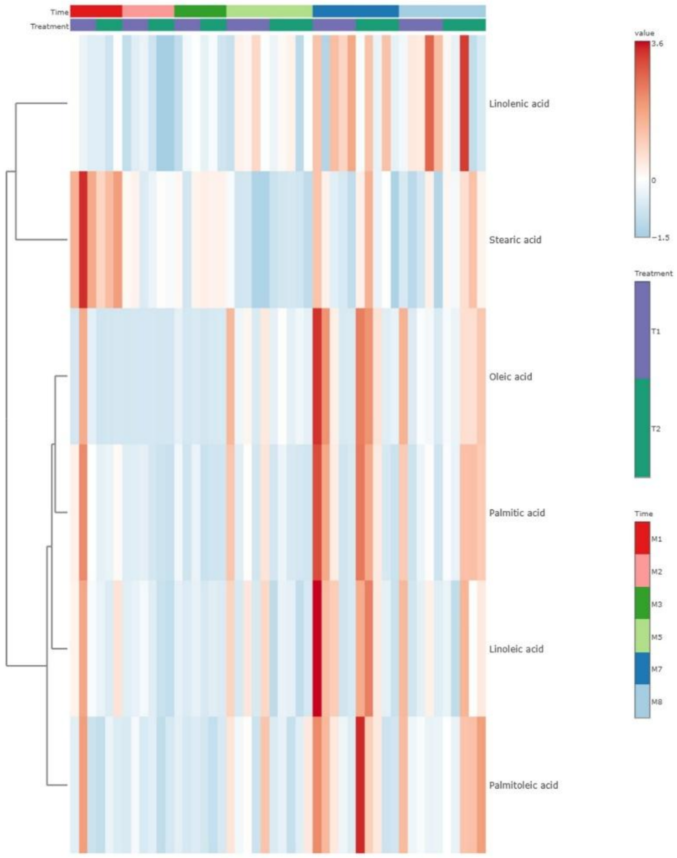

Figure S3. FAMEs profiles of the exocarp of avocado fruit. A) Principal Component Analysis (PCA) Score plot displaying the first two components and explained variance and B) Heatmap representation during different developmental stages (M1: February; M2: March; M3: May; M5: July; M7: September and M8: Harvest) and treatments (T1: 100% ETo and T2: 40% ETo). The columns represent biological replicates for each developmental stage and treatment, and the rows correspond to the metabolites. The similarity measure was used to cluster the different features was based on Euclidean distance and Ward's linkage from three or five biological replicates as detailed in the experimental design.

(A)

(B)

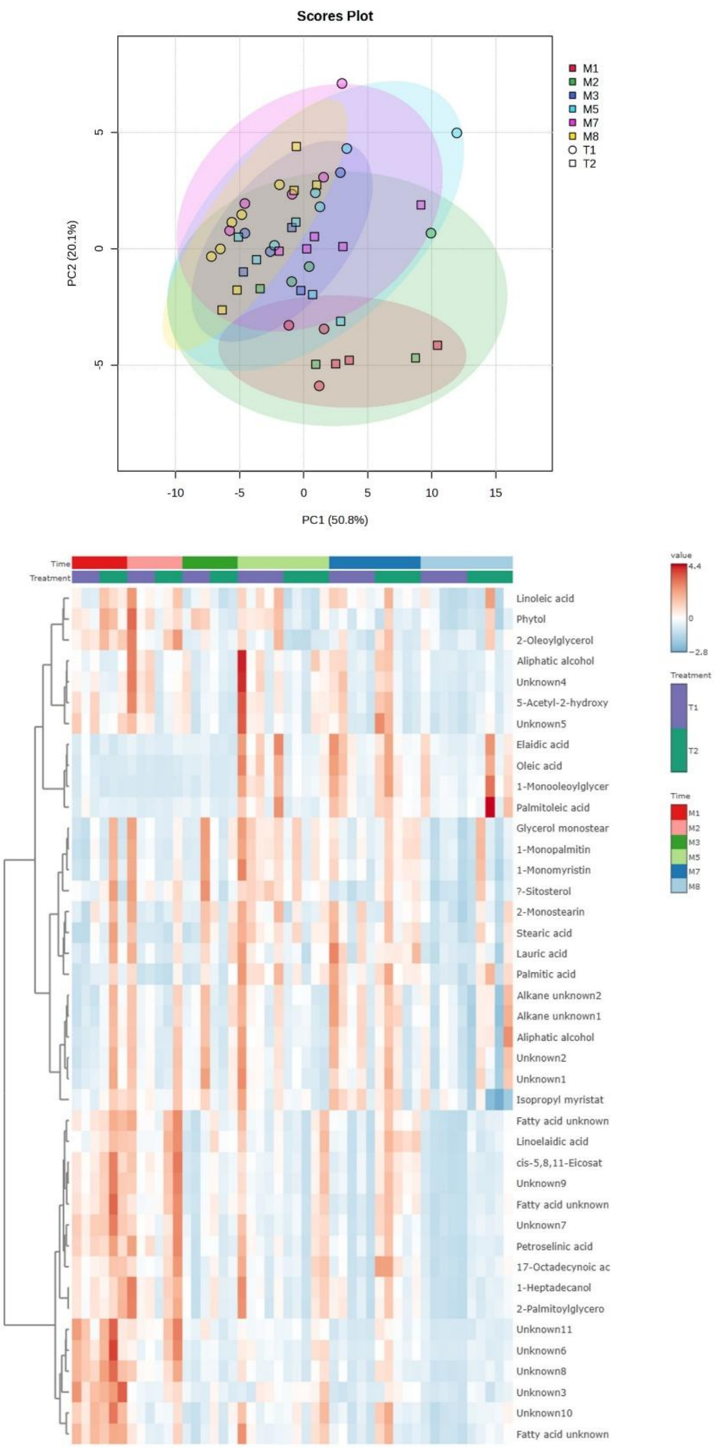

Figure S4. Nonpolar metabolite profiles of the exocarp of avocado fruit. A) Principal Component Analysis (PCA) Score plot displaying the first two components and explained variance and B) Heatmap representation during different developmental stages (M1: February; M2: March; M3: May; M5: July; M7: September and M8: Harvest) and treatments (T1: 100% ETo and T2: 40% ETo). The columns represent biological replicates for each developmental stage and treatment and the rows correspond to the metabolites. The similarity measure was used to cluster the different features was based on Euclidean distance and Ward's linkage from three or five biological replicates as detailed in the experimental design.
