# Supplementary material for: Effect of Water Regimen on Fruit Growth, Metabolomic Profile, and Postharvest Quality of ‘Hass’ Avocados
Source: Plants (Basel). 2026 Jun 11;15(12):1807. doi: 10.3390/plants15121807 (PMC13306940; doi:10.3390/plants15121807)
Supplement: Supplementary file 1 [file plants-15-01807-s001.zip › Supplementary Table S7 - Results pathway analysis.pdf]

Supplementary Table S7. Results from pathway analysis against *the Arabidopsis thaliana* pathway library

	Total Cmpd	Hits	Raw p	-log10(p)	Holm adjust	FDR	Impact
Fructose and mannose metabolism	18	1	3.09E-02	1.51E+00	1.00E+00	4.51E-01	0.07
Inositol phosphate metabolism	28	1	4.77E-02	1.32E+00	1.00E+00	4.51E-01	0.09
Ascorbate and aldarate metabolism	20	1	4.77E-02	1.32E+00	1.00E+00	4.51E-01	0.00
Glycerolipid metabolism	19	1	4.97E-02	1.30E+00	1.00E+00	4.51E-01	0.04
Galactose metabolism	27	4	5.03E-02	1.30E+00	1.00E+00	4.51E-01	0.05
Citrate cycle (TCA cycle)	20	4	5.25E-02	1.28E+00	1.00E+00	4.51E-01	0.22
Starch and sucrose metabolism	22	3	6.10E-02	1.21E+00	1.00E+00	4.51E-01	0.41
Valine, leucine and isoleucine degradation	37	2	8.63E-02	1.06E+00	1.00E+00	4.51E-01	0.00
Glyoxylate and dicarboxylate metabolism	29	9	8.65E-02	1.06E+00	1.00E+00	4.51E-01	0.29
Pyruvate metabolism	23	3	8.80E-02	1.06E+00	1.00E+00	4.51E-01	0.14
Amino sugar and nucleotide sugar metabolism	52	2	1.10E-01	9.59E-01	1.00E+00	4.51E-01	0.00
Carbon fixation by Calvin cycle	21	3	1.18E-01	9.29E-01	1.00E+00	4.51E-01	0.06
Valine, leucine and isoleucine biosynthesis	22	3	1.30E-01	8.87E-01	1.00E+00	4.51E-01	0.00
Pantothenate and CoA biosynthesis	25	1	1.49E-01	8.28E-01	1.00E+00	4.51E-01	0.00
Fatty acid biosynthesis	56	6	1.53E-01	8.16E-01	1.00E+00	4.51E-01	0.01
Tyrosine metabolism	17	1	1.55E-01	8.09E-01	1.00E+00	4.51E-01	0.10
Glucosinolate biosynthesis	65	3	1.61E-01	7.92E-01	1.00E+00	4.51E-01	0.00
Glycosylphosphatidylinositol (GPI)-anchor biosynthesis	28	1	1.64E-01	7.85E-01	1.00E+00	4.51E-01	0.05
Fatty acid elongation	23	1	1.64E-01	7.85E-01	1.00E+00	4.51E-01	0.00
Glycine, serine and threonine metabolism	33	5	1.78E-01	7.49E-01	1.00E+00	4.51E-01	0.55
D-Amino acid metabolism	7	1	2.01E-01	6.98E-01	1.00E+00	4.51E-01	0.00
Sphingolipid metabolism	27	1	2.01E-01	6.98E-01	1.00E+00	4.51E-01	0.00
Sulfur metabolism	12	1	2.01E-01	6.98E-01	1.00E+00	4.51E-01	0.00
Propanoate metabolism	20	1	2.04E-01	6.91E-01	1.00E+00	4.51E-01	0.00
Butanoate metabolism	17	3	2.14E-01	6.70E-01	1.00E+00	4.51E-01	0.14
One carbon pool by folate	21	2	2.19E-01	6.60E-01	1.00E+00	4.51E-01	0.02
Sesquiterpenoid and triterpenoid biosynthesis	24	1	2.39E-01	6.21E-01	1.00E+00	4.51E-01	0.00
Cysteine and methionine metabolism	46	2	2.49E-01	6.05E-01	1.00E+00	4.51E-01	0.00
Glycerophospholipid metabolism	38	2	2.49E-01	6.04E-01	1.00E+00	4.51E-01	0.03
Lipoic acid metabolism	24	1	2.52E-01	5.99E-01	1.00E+00	4.51E-01	0.00
Thiamine metabolism	22	1	2.52E-01	5.99E-01	1.00E+00	4.51E-01	0.00
Alanine, aspartate and glutamate metabolism	22	8	2.53E-01	5.97E-01	1.00E+00	4.51E-01	0.78
Phenylalanine, tyrosine and tryptophan biosynthesis	22	2	2.59E-01	5.87E-01	1.00E+00	4.51E-01	0.08
Fatty acid degradation	37	2	2.63E-01	5.80E-01	1.00E+00	4.51E-01	0.00
Arginine and proline metabolism	32	2	2.63E-01	5.80E-01	1.00E+00	4.51E-01	0.00
Cyanoamino acid metabolism	29	5	2.92E-01	5.35E-01	1.00E+00	4.68E-01	0.00
Cutin, suberine and wax biosynthesis	18	2	3.09E-01	5.10E-01	1.00E+00	4.68E-01	0.12
Pentose phosphate pathway	19	1	3.12E-01	5.06E-01	1.00E+00	4.68E-01	0.00
Monobactam biosynthesis	8	1	3.27E-01	4.85E-01	1.00E+00	4.68E-01	0.00
Lysine biosynthesis	9	1	3.27E-01	4.85E-01	1.00E+00	4.68E-01	0.00
beta-Alanine metabolism	18	1	3.27E-01	4.85E-01	1.00E+00	4.68E-01	0.00
Nicotinate and nicotinamide metabolism	13	1	3.27E-01	4.85E-01	1.00E+00	4.68E-01	0.00
Selenocompound metabolism	13	1	3.49E-01	4.57E-01	1.00E+00	4.78E-01	0.00
Glutathione metabolism	26	2	3.51E-01	4.55E-01	1.00E+00	4.78E-01	0.12
Arginine biosynthesis	18	4	3.64E-01	4.39E-01	1.00E+00	4.85E-01	0.13
Porphyrin metabolism	48	1	4.03E-01	3.95E-01	1.00E+00	5.26E-01	0.00
Biosynthesis of unsaturated fatty acids	22	5	4.28E-01	3.69E-01	1.00E+00	5.46E-01	0.00
Steroid biosynthesis	44	1	5.13E-01	2.90E-01	1.00E+00	6.28E-01	0.00
Brassinosteroid biosynthesis	26	1	5.13E-01	2.90E-01	1.00E+00	6.28E-01	0.00
Nitrogen metabolism	12	2	6.27E-01	2.03E-01	1.00E+00	7.52E-01	0.00
Pentose and glucuronate interconversions	17	2	6.61E-01	1.80E-01	1.00E+00	7.77E-01	0.00
Purine metabolism	75	1	7.46E-01	1.27E-01	1.00E+00	8.29E-01	0.00
Pyrimidine metabolism	41	1	7.46E-01	1.27E-01	1.00E+00	8.29E-01	0.00
Vitamin B6 metabolism	12	1	7.46E-01	1.27E-01	1.00E+00	8.29E-01	0.00
Phenylalanine metabolism	12	1	8.50E-01	7.06E-02	1.00E+00	8.95E-01	0.42
Phenylpropanoid biosynthesis	43	1	8.50E-01	7.06E-02	1.00E+00	8.95E-01	0.00
Tropane, piperidine and pyridine alkaloid biosynthesis	9	1	8.50E-01	7.06E-02	1.00E+00	8.95E-01	0.00
alpha-Linolenic acid metabolism	26	1	8.70E-01	6.07E-02	1.00E+00	9.00E-01	0.12
Glycolysis or Gluconeogenesis	26	1	9.30E-01	3.14E-02	1.00E+00	9.46E-01	0.00
Linoleic acid metabolism	4	1	9.85E-01	6.62E-03	1.00E+00	9.85E-01	1.00

Total is the total number of compounds in the pathway; *Hits* is the actually matched number from the user uploaded data; *Raw p* is the original p value calculated from the enrichment analysis; *Holm p* is the p value adjusted by Holm-Bonferroni method; *FDR p* is the p value adjusted using False Discovery Rate; *Impact* is the pathway impact value calculated from pathway topology analysis.
